# Case Report: Cerebral air embolism following fluid–air exchange during pars plana vitrectomy: a rare but severe complication

**DOI:** 10.3389/fnins.2026.1929675

**Published:** 2026-10-26

**Authors:** Rong Zhang, Guo Wen, Zhenqun Zhang

**Affiliations:** The First Affiliated Hospital of Hunan Traditional Chinese Medical College (Hunan Provincial Directly Affiliated Hospital of Traditional Chinese Medicine), Changsha, China

**Keywords:** acute cerebral infarction, cerebral air embolism, multidisciplinary intervention, pars plana vitrectomy, treatment

## Abstract

Cerebral air embolism (CAE) is a rare but potentially life-threatening neurological emergency, typically caused by the inadvertent entry of exogenous air into the venous circulation and subsequent migration to the cerebral vasculature. It presents with abrupt onset and a prognosis that heavily depends on early recognition and prompt intervention. We report a case of CAE that occurred during pars plana vitrectomy (PPV) involving intraoperative fluid–air exchange. The patient experienced sudden ocular pain intraoperatively, followed shortly post-operatively by acute neurological symptoms including altered consciousness, ataxia, and slurred speech. Brain MRI using a standard stroke protocol, including DWI with b values of 0 and 1,000 s/mm^2^ and ADC maps, revealed multiple acute infarcts in the bilateral frontal, parietal, and occipital lobes, thalamus, and corpus callosum. DWI demonstrated widespread hyperintensity with corresponding ADC reduction. Although no definite intravascular air artifacts were detected, the combination of intraoperative pressure fluctuations, a hypercoagulable state, and elevated levels of D-dimer and cardiac troponin I strongly supported the diagnosis of CAE with possible concomitant microembolism-induced ischemia. The patient received prompt multidisciplinary intensive care and exhibited marked neurological recovery with favorable prognosis. This case highlights the need for high clinical suspicion of CAE during procedures involving air infusion, abrupt pressure changes, or vascular injury risk. Emergent neuroimaging and multidisciplinary management are critical to minimizing neurological sequelae and ensuring patient safety.

## Introduction

Pars plana vitrectomy (PPV) is a widely used surgical technique for the treatment of rhegmatogenous retinal detachment, vitreous hemorrhage, and macular pathologies. It is favored for its minimally invasive nature and rapid post-operative recovery (McCarthy et al., 2017). However, despite continuous advancements in surgical techniques and instrumentation, intraoperative and post-operative complications such as choroidal hemorrhage, recurrent retinal detachment, and intraocular pressure dysregulation can still occur (Muller et al., 2021; Marsh et al., 2023). Expulsive suprachoroidal hemorrhage (ESCH) remains a rare but catastrophic event, characterized by abrupt onset and rapid progression, which can result in irreversible ocular damage or even loss of the globe if not managed promptly (Brito et al., 2020). Importantly, recent studies have drawn attention to an even rarer but potentially fatal complication during intraocular surgeries—air embolism. In particular, when procedures involve sudden posterior segment pressure shifts, inadvertent venous injury, or infusion line mishandling, there is a theoretical risk of air entering the venous system and reaching the central nervous system (CNS), leading to acute neurological events such as cerebral infarction, seizures, or altered consciousness (Bellucci et al., 2023; Zhang et al., 2022). Ocular venous air embolism (OVAE) is a proposed mechanism, wherein the dislodgment of infusion cannulas during PPV may result in gas entering the suprachoroidal space, subsequently tearing vortex veins and allowing air to enter the systemic circulation (Mo et al., 2021). The risk is further elevated in cases involving trauma, tumor excision (e.g., choroidal melanoma), or significant vascular exposure (Zhang et al., 2022). OVAE often presents insidiously, with sudden respiratory or cardiovascular collapse being the initial manifestation, followed in some cases by CNS complications.

In this study, we present a case of a 61-year-old woman who developed ESCH during vitrectomy for rhegmatogenous retinal detachment, followed shortly by acute neurological deterioration. Imaging confirmed multiple acute cerebral infarctions. Based on intraoperative events, radiological findings, and review of the literature, we suspect intraoperative gas embolism or microembolism as the likely etiology. The patient made a favorable recovery with timely recognition and multidisciplinary intervention. This case underscores the need for heightened vigilance regarding systemic complications beyond the ocular field during intraocular surgeries, and provides valuable insight into the clinical recognition and management of intraoperative air embolism.

## Case

A 61-year-old woman was admitted to the Department of Ophthalmology on April 7, 2025, with a 7-day history of decreased vision and visual shadowing in her right eye. Her past medical history was notable only for cataract surgery in the right eye (phacoemulsification and intraocular lens implantation in 2020), with good post-operative recovery. She had no history of hypertension, diabetes, stroke, arrhythmia, thrombotic diseases, smoking, alcohol use, or hereditary disorders. On admission, physical examination revealed no light perception in the right eye and a visual acuity of 0.6 in the left eye. The anterior segment of the right eye was unremarkable, with normal intraocular pressure. Fundus examination revealed a rhegmatogenous retinal detachment (RRD) extending to the macular region, with a horseshoe tear located at the 5 o'clock position. Optical coherence tomography (OCT), scanning laser ophthalmoscopy (SLO), and B-scan ultrasonography confirmed the diagnosis. PPV combined with laser photocoagulation and perfluorocarbon liquid (PFCL) injection was planned. On April 8, under local anesthesia, the patient underwent standard three-port PPV with subretinal fluid drainage. After injection of heavy liquid, the retina was successfully reattached. During preparation for silicone oil tamponade, the patient suddenly complained of severe ocular pain accompanied by profuse sweating and a near-death sensation. Pulse oximetry revealed a transient drop in oxygen saturation to 88%. A dome-shaped choroidal elevation was observed intraoperatively, raising suspicion for ESCH. The procedure was immediately halted, retaining only 3 mL of PFCL in the vitreous cavity. The sclerotomies were closed, and intravenous hemostatic agents (hemocoagulase and tranexamic acid) were administered. Estimated intraoperative blood loss was 2 mL, limited to the posterior segment without anterior chamber involvement or globe rupture. Approximately 1 h post-operatively, the patient developed sudden onset of altered mental status, slurred speech, and gait instability, rapidly progressing to inability to ambulate independently, raising suspicion for a CNS event. Emergent brain MRI was performed on a 1.5-T scanner using a standardized stroke protocol, including axial T1WI, T2WI, FLAIR, DWI with b values of 0 and 1,000 s/mm^2^, corresponding ADC maps. Section thickness was 4 mm with a 1 mm interslice gap, and no contrast agent was administered. DWI revealed multiple hyperintense lesions in the bilateral frontal, parietal, and occipital lobes, as well as the right thalamus and corpus callosum, with corresponding hypointensity on ADC maps, consistent with acute infarcts. FLAIR showed corresponding hyperintensity at representative levels (Figure 1). No definite intracranial air was identified within the veins, venules, capillaries, or other intracranial compartments on the available MRI sequences or on CT angiography. Cranio-cervical CTA showed no large-vessel occlusion or stenosis, and pulmonary CTA showed no evidence of pulmonary embolism (Figure 2). All MRI and CTA images were acquired using a standardized stroke protocol and were reviewed by a radiologist with experience in neuroimaging. Laboratory tests showed elevated D-dimer (932 μg/L) and cardiac troponin I (cTnI, 2.981 ng/mL). Arterial blood gas analysis revealed mild hypoxemia (PO_2_: 72 mmHg). Electrocardiogram and echocardiography showed no signs of myocardial ischemia. Given the intraoperative hemodynamic stress, abnormal venous return, and suspected orbital hypertension due to hemorrhage, a diagnosis of cerebral air embolism (CAE) or microembolic cerebral infarction was strongly suspected. Hyperbaric oxygen therapy was initiated (April 9, 19:30–22:50), followed by ICU admission for management including intracranial pressure reduction, neuroprotection, antiplatelet and anticoagulant therapy, cardioprotection, and supportive care. The patient's neurological status improved progressively; by day 10, she was ambulatory and had fully recovered her speech and cognition. On post-operative day 11, under general anesthesia, she underwent successful completion surgery with PFCL removal and silicone oil tamponade. No further complications occurred. At discharge, her right eye visual acuity improved to hand motion. At 6-month follow-up, she remained neurologically stable with a National Institutes of Health Stroke Scale (NIHSS) score of 0 and a modified Rankin Scale (mRS) score of 1. Follow-up OCT demonstrated a well-attached retina.

**Figure 1:**
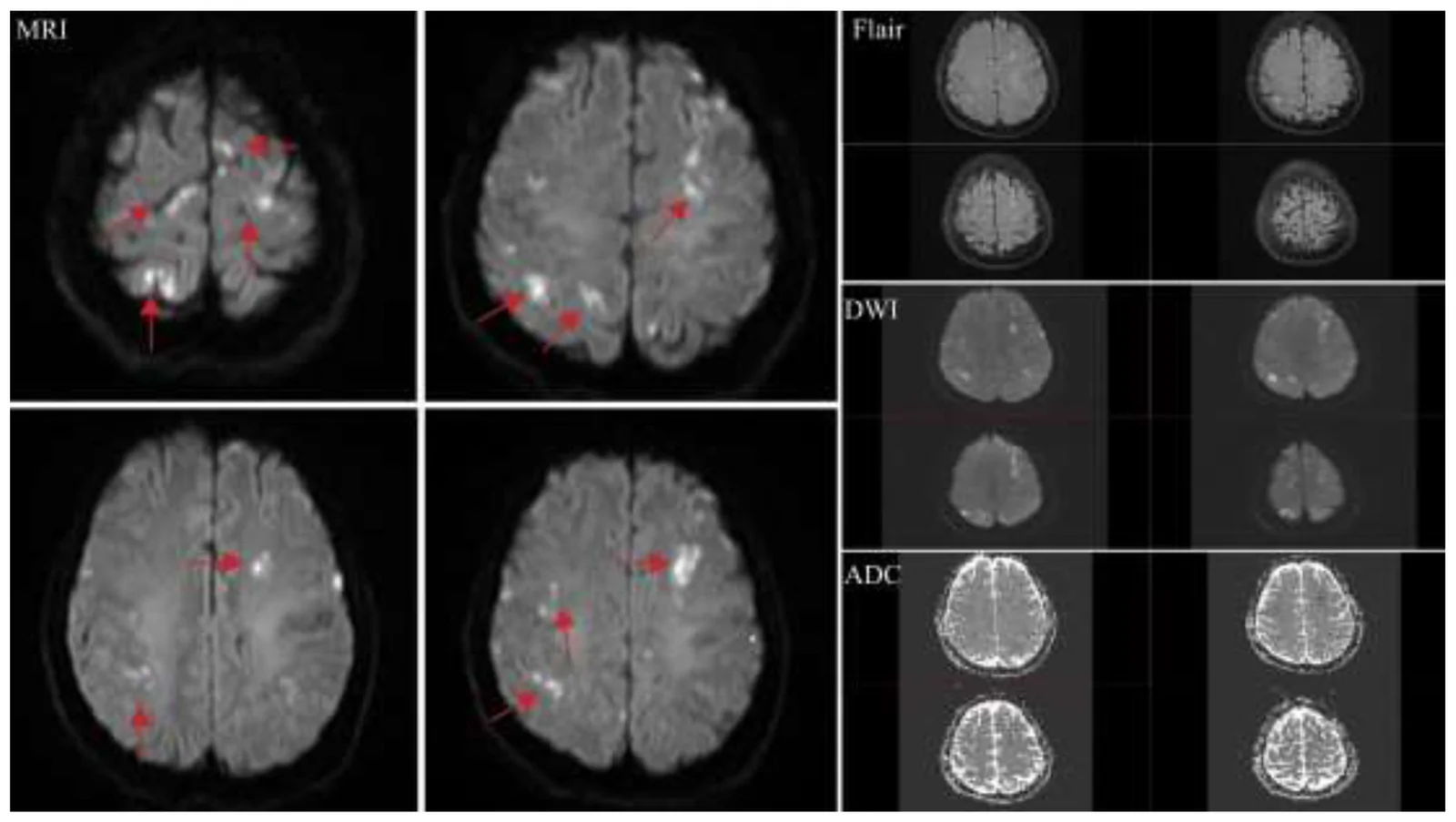
Diffusion-weighted MRI performed approximately 1 h post-operatively revealed multiple acute infarcts in the bilateral frontal, parietal, and occipital lobes, as well as the right thalamus and corpus callosum. These lesions appeared as hyperintense signals on DWI (highlighted by red arrows), consistent with subcortical acute ischemic infarctions. Axial diffusion-weighted imaging (DWI, b = 1,000 s/mm^2^) shows multiple hyperintense lesions in the bilateral frontal, parietal, and occipital lobes (arrows). Corresponding apparent diffusion coefficient (ADC) map shows hypointensity in the same regions, consistent with acute infarcts. Axial fluid-attenuated inversion recovery (FLAIR) image at a representative level shows corresponding hyperintensity.

**Figure 2:**
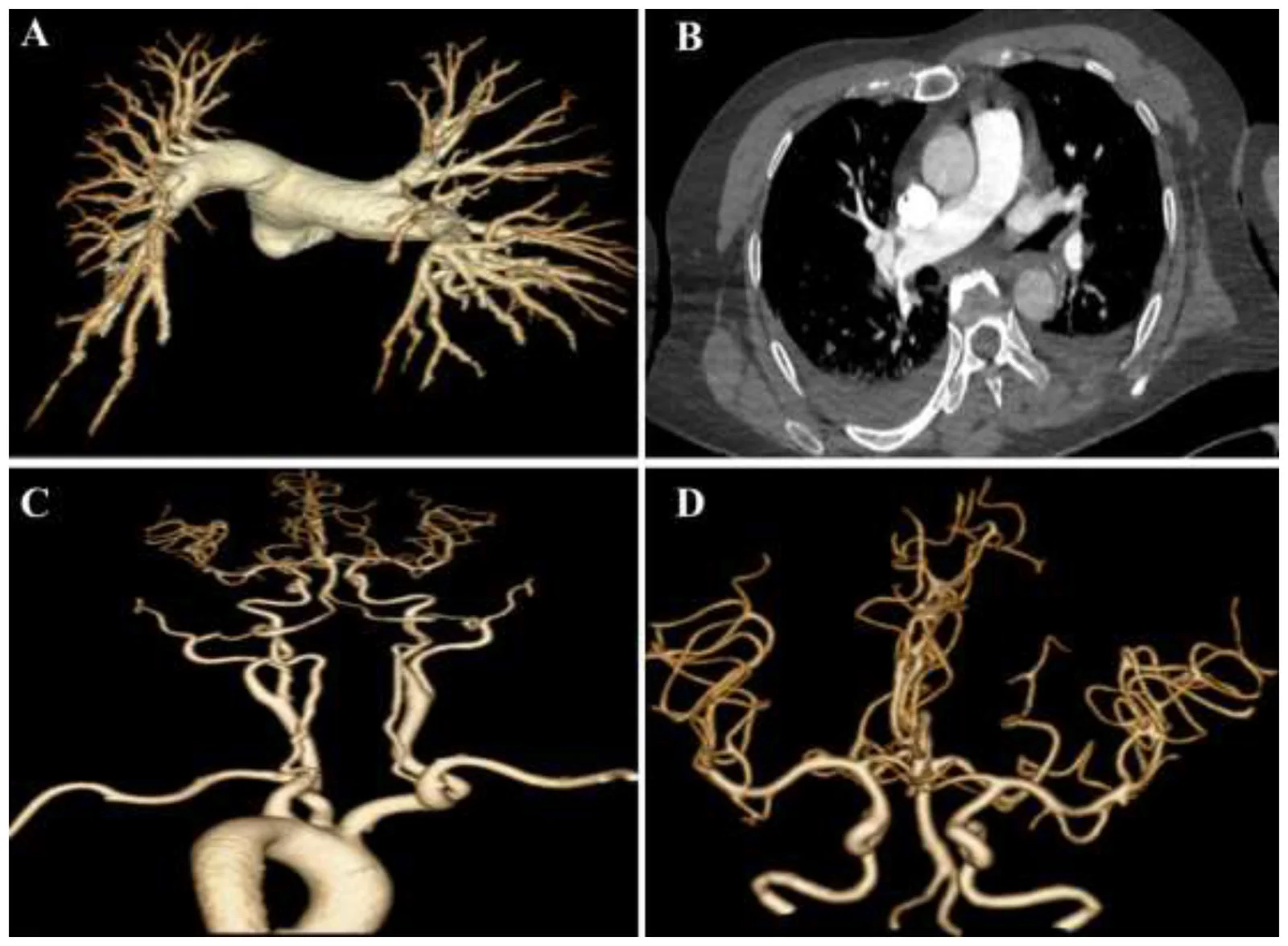
CT angiography (CTA) findings in a patient with suspected cerebral air embolism after pars plana vitrectomy. **(A)** Three-dimensional volume-rendered reconstruction of the pulmonary arteries shows patent bilateral pulmonary arterial trees without filling defects, consistent with the absence of pulmonary embolism. **(B)** Axial contrast-enhanced pulmonary CTA demonstrates a normal pulmonary trunk and main branches without intraluminal filling defects. **(C, D)** Cranio-cervical CT angiography shows patent cervical and intracranial arteries without large-vessel occlusion or stenosis, displayed in anteroposterior **(C)** and lateral/oblique **(D)** projections for anatomical orientation.

## Discussion

The novelty of this case lies in the rare occurrence of cerebral air embolism during pars plana vitrectomy complicated by expulsive suprachoroidal hemorrhage, and in the characteristic pattern of multiple small subcortical infarcts without large-vessel occlusion. This combination has rarely been reported and highlights the need to consider systemic embolic complications during intraocular surgery, even when intravascular air is not directly visualized on imaging. Table 1 summarizes the key features of previously reported cases of ocular venous air embolism and choroidal hemorrhage during vitrectomy, alongside the present case. Unlike prior reports that focused primarily on ocular outcomes, the present case is notable for the development of multiple acute cerebral infarcts following an intraoperative pressure event, underscoring the need for systemic vigilance during intraocular surgery.

**Table 1:** Comparison of the present case with previously reported cases of OVAE or CAE during vitrectomy.

Feature	Present case (2025)	Mo et al. (2021)	Bellucci et al. (2023)	Zhang et al. (2022)
Trigger event	PPV + ESCH during PFCL injection; suspected suprachoroidal air infusion	Suprachoroidal hemorrhage during PPV	PPV combined with phacoemulsification	Intraoperative choroidal detachment during small-gauge vitrectomy
Latency to neurological symptoms	~1 h post-operatively	N/A (ocular focus)	N/A (ocular focus)	N/A (ocular focus)
Imaging pattern	Multiple acute infarcts (DWI hyperintensity/ADC hypointensity) in bilateral frontal, parietal, occipital lobes, thalamus, corpus callosum	Choroidal hemorrhage on ultrasound/CT	Retinal detachment assessment	Choroidal detachment on intraoperative observation
Treatment	100% oxygen, HBOT, ICU, antiplatelet/anticoagulant, neuroprotection	Hemostatic agents, surgery halted	Surgical management	Surgical management
Outcome	Full neurological recovery (NIHSS 0, mRS 1); retina attached	Variable visual outcomes	Favorable retinal outcomes	Variable visual outcomes

An important differential or compounding mechanism in this case is paradoxical arterial embolism through a right-to-left shunt. The bilateral, multi-territory “shower” pattern of infarcts on DWI is not fully explained by a purely venous-to-pulmonary route, and raises the possibility that venous gas bubbles bypassed the pulmonary capillary filter through a patent foramen ovale (PFO) or another right-to-left shunt, or through transpulmonary passage when the pulmonary clearance capacity was exceeded (Moon, 2019; Moon and Mitchell, 2025). In the context of pars plana vitrectomy, even a small volume of venous air entering the systemic circulation could reach the cerebral microvasculature through such a shunt, producing scattered ischemic lesions. Transesophageal echocardiography (TEE) with bubble-contrast study, or transcranial Doppler (TCD) with agitated saline, is the preferred approach to detect a right-to-left shunt, and its early use should be considered in similar cases. In the present patient, however, TEE and TCD were not performed during the acute event, so a right-to-left shunt could not be confirmed or excluded, this is acknowledged as a limitation.

Air embolism can enter the systemic circulation through various mechanisms. CAE is a rare but life-threatening neurological emergency characterized by sudden onset and rapid progression. Its prognosis is highly dependent on the extent of vascular involvement and the timeliness of medical intervention (Červeňák et al., 2024). Cerebral air embolism most commonly occurs after cardiothoracic or interventional procedures. Once CAE occurs, interruption of cerebral perfusion leads to ischemic injury, typically manifesting as altered consciousness, seizures, or coma (Adachi et al., 2025). Imaging findings are often non-specific, especially as small gas bubbles may be rapidly resorbed and fail to appear on CT scans, making clinical suspicion and contextual judgment essential for diagnosis. PPV involves the intraocular infusion of exogenous gas or fluids—such as air, perfluorocarbon liquids (PFCL), and silicone oil—to maintain intraocular structure and retinal positioning. Under circumstances of infusion line malfunction, abrupt posterior segment pressure fluctuations, or rupture of choroidal vessels, the risk of air embolism, though rare, cannot be entirely excluded (Huang et al., 2024). In the present case, the patient developed acute CNS symptoms—including confusion, gait instability, and slurred speech—within 1 h post-operatively. Brain MRI revealed multiple acute infarcts involving the bilateral frontal, parietal, and occipital lobes, thalamus, and corpus callosum, primarily in a subcortical distribution. The temporal proximity to surgery, combined with the intraoperative occurrence of ESCH, elevated D-dimer levels, and a marked rise in cardiac troponin I (cTnI) in the absence of myocardial ischemia, strongly support the clinical suspicion of CAE or microembolism-related cerebral infarction. Intraoperative ESCH causes abrupt IOP elevation, which may promote retrobulbar venous congestion and allow small gas bubbles to enter the ocular venous system and reach the intracranial venous circulation. The proposed mechanism is dislodgement or slippage of the infusion cannula, causing suprachoroidal pressurized-air infusion, which tears the vortex veins and allows air entrainment into the valveless ocular venous system (Morris et al., 2019). The anatomical route of OVAE is well-described: air enters the vortex veins, drains into the superior and inferior ophthalmic veins, and then passes through the cavernous sinus into the jugular vein and right atrium (Morris et al., 2019). Because these veins are valveless, air can travel along pressure gradients in a retrograde or antegrade fashion, reaching the intracranial venous system and, in the presence of a right-to-left shunt, the systemic arterial circulation (Marsh et al., 2023). This pathway explains why the infarcts in this patient were multifocal and bilateral rather than confined to a single vascular territory. Once within cerebral venous microcirculation—particularly in subcortical regions—these microbubbles, though minimal in volume, may disrupt local hemodynamics and provoke vasoreactive ischemia. Importantly, such gas bubbles are often rapidly absorbed by plasma and may be radiologically “invisible,” making DWI the most sensitive modality to detect resultant ischemic injury. In this case, the absence of visible intracranial air on imaging does not preclude the diagnosis of air embolism. An additional mechanism that may contribute to cerebral air embolism is paradoxical air embolism. In the presence of a right-to-left shunt, such as a patent foramen ovale or pulmonary arteriovenous malformation, venous gas bubbles can bypass the pulmonary capillary filter and enter the systemic arterial circulation, potentially causing cerebral or coronary ischemia. Paradoxical air embolism may also occur through transpulmonary passage of gas when the pulmonary clearance capacity is exceeded. In the context of pars plana vitrectomy, even a small volume of venous air entering the systemic circulation could reach the cerebral microvasculature through such a shunt, producing scattered ischemic lesions. This mechanism is particularly relevant in patients with undiagnosed intracardiac shunts, and it may explain the pattern of multiple small infarcts observed in our patient. A recent case report and literature review on retrograde venous cerebral air embolism infarcts provides further insight into the radiological and clinical features of this condition (Teifurova et al., 2025). Even minimal air emboli may result in disseminated subcortical ischemic lesions via microvascular obstruction or reactive vasospasm. The pattern of multiple small infarcts in this patient can be explained by the behavior of small gas bubbles in the cerebral microcirculation. When microbubbles enter the cerebral vasculature, they are distributed diffusely across subcortical and cortical microvascular beds rather than occluding a single large vessel. These bubbles can obstruct capillaries and venules, trigger local vasospasm, activate platelets and the coagulation cascade, and induce endothelial injury, collectively producing scattered ischemic lesions. In addition, the hypercoagulable state suggested by elevated D-dimer and cardiac troponin I levels in this case may have further promoted microthrombus formation and amplified the ischemic burden. This mechanism is consistent with the small, multiple, bilateral, and subcortical distribution of infarcts observed in our patient, and also explains why no large-vessel occlusion was identified on CTA. Therefore, in intraocular surgeries complicated by significant IOP changes or infusion abnormalities—especially when followed by acute neurological deterioration—CAE should be considered a high-priority differential diagnosis, even in the absence of radiologically detected gas. Prompt recognition and multidisciplinary intervention are crucial to minimizing neurological sequelae.

In addition to the possible contribution of air embolism, a hypercoagulable state induced by intraoperative physiological stress may have also played a role in this case. PPV is often the second ocular procedure in elderly patients, and intraoperative factors such as psychological stress, nociceptive stimulation, and abrupt IOP elevation can activate the sympathetic nervous system. This, in turn, may lead to vasoconstriction, platelet activation, and increased procoagulant activity. Post-operative local inflammation can further exacerbate coagulation factor elevation. Although non-specific, the markedly elevated levels of D-dimer and cTnI observed in this case suggest systemic inflammatory responses and endothelial dysfunction—both of which are recognized risk factors for microvascular occlusion (Kim et al., 2019). Therefore, the cerebral infarction observed in this patient was likely the result of a multifactorial process, including retrobulbar hypertension induced by ESCH, residual microbubbles in the infusion system, and intraoperative stress-mediated microthrombus formation. This complex pathophysiological cascade highlights the need to look beyond the local surgical field when considering complications following intraocular procedures, and instead to anticipate and mitigate potential systemic embolic risks. The management pathway in this case also provides a useful reference for similar clinical scenarios. In any patient presenting with acute neurological symptoms shortly after ophthalmic surgery, prompt initiation of neuroimaging is essential. DWI should be prioritized to detect scattered subcortical infarcts or venous outflow obstruction. Concurrent laboratory assessments—including D-dimer, cTnI, electrocardiogram (ECG), and echocardiography—are recommended to evaluate for possible cardiogenic or thrombotic causes. If the clinical context, intraoperative events, and imaging findings suggest air or microembolic mechanisms, early admission to the intensive care unit (ICU) should be considered for comprehensive management. This includes neuroprotection, antiplatelet and anticoagulant therapy, and intracranial pressure control. In this case, timely multidisciplinary intervention enabled the patient to achieve full recovery of ambulation and speech by post-operative day 10. Final follow-up showed stable retinal attachment on OCT, with a NIHSS score of 0 and a mRS score of 1—strongly supporting the clinical value of coordinated cross-specialty care.

In patients with acute neurological deterioration shortly after intraocular surgery, urgent neuroimaging should follow a standardized acute stroke imaging algorithm. The first-line MRI protocol should include diffusion-weighted imaging with b values of 0 and 1,000 s/mm^2^ and corresponding apparent diffusion coefficient maps, which are most sensitive for acute ischemic lesions. Fluid-attenuated inversion recovery and T2-weighted imaging help assess lesion extent and exclude other structural causes. Susceptibility-weighted imaging or T2^*^-weighted gradient-echo imaging should be included to detect intracranial air or hemorrhage. Magnetic resonance angiography is recommended to exclude large-vessel occlusion and cerebral venous sinus thrombosis. If MRI is unavailable or the patient is unstable, non-contrast head CT may be used for initial triage, although it is less sensitive for acute ischemia and may miss small gas bubbles. When cerebral air embolism is suspected, the absence of visible intravascular air does not exclude the diagnosis, and diffusion-weighted imaging should be performed or repeated promptly. In parallel, laboratory tests including D-dimer, cardiac troponin I, arterial blood gas analysis, electrocardiography, and echocardiography should be obtained. This algorithm facilitates early recognition of cerebral air embolism or microembolic infarction and supports timely multidisciplinary intervention.

When CAE is suspected or confirmed, immediate systemic intervention is essential (Silva et al., 2022). The first-line clinical measure is the administration of 100% high-flow oxygen, which facilitates the diffusion of nitrogen out of the air bubbles, accelerates bubble resorption, and improves tissue oxygenation. In patients with significant neurological involvement, early initiation of hyperbaric oxygen therapy (HBOT) is recommended. HBOT increases the partial pressure of oxygen in the blood and reduces bubble volume, thereby mitigating cerebral ischemia and secondary edema. When initiated within 6 h of symptom onset, HBOT has been shown to significantly improve neurological outcomes. In parallel, it is crucial to maintain stable hemodynamics through appropriate fluid resuscitation while avoiding hypotension, in order to preserve adequate cerebral perfusion. Based on individual risk assessment, antiplatelet or anticoagulant therapy may be considered to prevent or treat microthrombus formation. A supine position is generally preferred to minimize intracranial pressure. In patients with radiological evidence of cerebral infarction, neuroprotective strategies—including osmotic agents for intracranial pressure reduction, glucose and temperature control—should be promptly implemented. In this case, the patient received timely HBOT and supportive care under the coordination of a multidisciplinary team. Prompt recognition and early initiation of oxygen therapy and antithrombotic treatment resulted in full neurological recovery. This underscores the critical importance of early diagnosis and rapid, targeted intervention in the effective management of CAE.

An important practical determinant of outcome in iatrogenic CAE is timely access to HBOT. In real-world settings, the availability of a hyperbaric chamber, rather than the clinical indication itself, often dictates whether treatment can be delivered within the therapeutic window. Current evidence-based recommendations support early HBOT for patients with significant neurological involvement, with the greatest benefit when treatment is initiated within 6 h of symptom onset (Moon and Mitchell, 2025). However, this evidence is derived primarily from observational studies, case series, and expert consensus rather than randomized controlled trials (Moon, 2019; Moon and Mitchell, 2025). As illustrated by Thomas et al., patients may require transfer to alternative facilities, including outpatient hyperbaric centers, when immediate access is not available at the treating hospital (Thomas et al., 2025). This logistical reality underscores the need for early identification of HBOT-capable centers and proactive coordination of transfer when CAE is suspected.

The management of thrombotic cerebrovascular disease offers instructive parallels, but also important distinctions. Air emboli are compressible and largely resorb, so mechanical thrombectomy devices designed for solid clot do not directly apply to CAE. However, the stroke code model of rapid multidisciplinary triage and standardized imaging-to-treatment intervals provides a useful structural comparator for organizing the acute response to suspected CAE. A closer anatomical parallel may be cerebral venous sinus thrombosis (CVST), in which the proposed air route in this case terminates. In CVST, anticoagulation is first-line therapy, while endovascular thrombectomy is reserved for patients' refractory to or ineligible for anticoagulation (Kan et al., 2024). This paradigm supports the use of anticoagulation, rather than mechanical intervention, as the appropriate initial strategy in this patient, in whom the proposed mechanism involves venous-to-cavernous sinus air transmission with secondary microvascular ischemia.

The true incidence of ocular venous air embolism and cerebral air embolism during ophthalmic procedures is likely underestimated. Only 13 cases of OVAE have been reported in the literature, yet 69% of these were fatal, suggesting that fatal cases are more likely to be published while non-fatal or aborted events remain unreported (Morris et al., 2019). A survey of vitreoretinal surgeons found that only 20% were aware of OVAE as a potential complication, and many cannula displacements are likely stopped before cardiovascular collapse occurs and therefore never reported (Morris et al., 2019). Similar patterns of underreporting have been recognized in other embolization contexts. A recent systematic review and meta-analysis of middle meningeal artery embolization explicitly noted “variability in adverse event reporting” and “insufficient documentation of rare complications” across the included studies (Fahim et al., 2026). While this observation comes from a different embolization procedure, it illustrates a cross-cutting methodological problem: rare but clinically important adverse events are systematically under-captured in retrospective cohorts and single-center series. By analogy, CAE following pars plana vitrectomy is likely underrecognized and underreported, and the absence of large case series should not be interpreted as evidence of negligible risk.

This case has several limitations. First, it is a single-case report, and the findings cannot be generalized to a broader population. Second, the intraoperative pressure event was reconstructed retrospectively from surgical records and clinical observations, rather than measured prospectively, which limits the precision of the proposed mechanism. Third, transesophageal echocardiography and transcranial Doppler were not performed during the acute event, so a right-to-left shunt or paradoxical embolism could not be confirmed or excluded. Fourth, the diagnosis of cerebral air embolism was inferential rather than radiographically confirmed, because no definite intravascular air artifacts were detected on the available MRI sequences or on CT angiography. An alternative explanation, namely stress-related microthrombosis in the setting of a hypercoagulable state, cannot be entirely excluded. These limitations should be considered when interpreting our findings.

In conclusion, although PPV is considered a routine ophthalmic procedure, the potential risk of air embolism and secondary cerebral infarction should not be overlooked, especially in the presence of intraoperative hemorrhage, fluid infusion, or structural disruption. Moving forward, clinical practice must place greater emphasis on pre-operative risk assessment, careful intraoperative gas and fluid management, maintenance of wound integrity, and rapid recognition of post-operative neurological abnormalities. This case highlights the need to shift the clinical mindset from ensuring “intraocular safety” alone to safeguarding “systemic safety” as a whole. The rare and complex progression observed in this patient not only reveals the true risk of uncommon complications associated with PPV but also provides valuable insights for the timely diagnosis and stratified management of post-operative neurological events.

## Data Availability

The original contributions presented in the study are included in the article/supplementary material, further inquiries can be directed to the corresponding author.
